# Risk and Resilience Factors Related to Parental Bereavement Following the Death of a Child with a Life-Limiting Condition

**DOI:** 10.3390/children4110096

**Published:** 2017-11-09

**Authors:** Tiina Jaaniste, Sandra Coombs, Theresa J. Donnelly, Norm Kelk, Danielle Beston

**Affiliations:** 1 Department of Pain and Palliative Care, Sydney Children’s Hospital, Randwick NSW 2031, Australia; Sandra.Coombs@health.nsw.gov.au (S.C.); TheresaJDonnelly@gmail.com (T.J.D.); Norm.Kelk@health.nsw.gov.au (N.K.); Danielle.Beston@health.nsw.gov.au (D.B.); 2 School of Women’s and Children’s Health, University of New South Wales, Kensington NSW 2052, Australia

**Keywords:** bereavement, palliative care, parents, risk factors, resilience factors

## Abstract

This paper reviews the theoretical and empirical literature on risk and resilience factors impacting on parental bereavement outcomes following the death of a child with a life-limiting condition. Over the past few decades, bereavement research has focussed primarily on a risk-based approach. In light of advances in the literature on resilience, the authors propose a Risk and Resilience Model of Parental Bereavement, thus endeavouring to give more holistic consideration to a range of potential influences on parental bereavement outcomes. The literature will be reviewed with regard to the role of: (i) loss-oriented stressors (e.g., circumstances surrounding the death and multiple losses); (ii) inter-personal factors (e.g., marital factors, social support, and religious practices); (iii) intra-personal factors (e.g., neuroticism, trait optimism, psychological flexibility, attachment style, and gender); and (iv) coping and appraisal, on parental bereavement outcomes. Challenges facing this area of research are discussed, and research and clinical implications considered.

## 1. Introduction

Bereavement, or the loss of a loved one through death, results in a process of adaptation to living following the death of the loved one [1]. The grief, or distress resulting from bereavement experienced by a parent following the death of a child is widely recognized as one of the most intense and persistent types of bereavement [2]. Although the main cause of death in childhood is trauma (unexpected accidents and injuries), a much smaller proportion of childhood deaths is due to life-limiting conditions, i.e., conditions which may significantly reduce the child’s life-span, with no reasonable hope of cure. Parental bereavement that follows weeks, months or years of caring for a child with a life-limiting condition is a particular context that is likely to have a unique set of factors associated with parental bereavement outcomes. Following a brief overview of some of the unique aspects of the bereavement experience associated with the death of a child due to a life-limiting condition, the current narrative review will consider a range of factors that may influence parental adjustment in this context. The Integrative Risk Factor Framework of Bereavement by Stroebe et al. [3] provides a good structure for considering a broad array of possible risk factors that may impact on the ability of parents to resume functioning with valued activities following the death of their child. The current paper extends the framework of Stroebe et al. [3] to also incorporate a consideration of protective or resilience factors. Thus, the literature pertaining to a broad range of possible risk and resilience factors will be reviewed from within the context of our newly proposed Risk and Resilience Model of Parental Bereavement, with particular focus on the bereavement context following the death of a child with a life-limiting condition. The current narrative review is based on an extensive (though not systematic) review of the literature. Where possible, the associations between risk and resilience factors with bereavement outcomes have been considered in terms of the levels of evidence. Some of the challenges and limitations of carrying out research in this area will be discussed, and implications for further research and clinical practice considered.

## 2. Parental Bereavement Following the Death of a Child with a Life-Limiting Condition

There are a number of key differences between parental bereavement processes associated with the death of a child with a prolonged, life-limiting condition, relative to an unexpected death due to an injury or acute illness [4,5]. Parents looking after a child with a life-limiting condition commonly experience prolonged suffering in the weeks, months, and years before the death of the child. In some cases, they may have learnt about their child’s diagnosis around the time of their birth. The child’s death may occur at a point when a parent’s coping resources have been tested to the limit across a range of possible areas (e.g., physical, emotional, marital, financial, spiritual, and inter-personal), often for a long period of time. On the one hand, the draining nature of a prolonged period of caring may leave an individual vulnerable to poorer bereavement outcomes. Conversely, however, the stress reduction theory holds that the child’s death coincides with a reduction in stressors that had been associated with the long-term care of the child, thus potentially facilitating post-bereavement adjustment [6]. It is extremely difficult to compare bereavement outcomes for expected and unexpected child deaths in a methodologically rigorous way, and results have been mixed. One study found lower levels of parental depression following an unexpected death relative to a long-term illnesses [7], whereas another study found poorer parental bereavement outcomes following a child’s violent death (e.g., resulting from accident, homicide, and suicide) relative to a long-term illness [8].

In the context of caring for a child with a life-limiting condition, parental grief processes may have started well before the child’s actual death, with similarities (albeit also some differences) between anticipatory mourning/grief reactions and conventional grief reactions [9]. Upon learning of a child’s diagnosis and/or prognosis, the parents of a child with a life-limiting condition may start to readjust their cognitive schemas, to accommodate the recognition that their child might not experience a “normal” childhood, nor live to adulthood. However, this recognition does not necessarily equate with preparedness. Indeed, this realization may be associated with such a burden to parents that some authors have described the potential for the draining and debilitating experience of “chronic sorrow” [10].

The parents of children with a life-limiting condition may be more likely than the parents of children following an injury or acute illness to be aware if their child’s impending death is imminent. Parents commonly report that the knowledge of their child’s impending death enabled them to make appropriate choices, engage in tasks they deemed most important prior to their child’s death, and to say goodbye to their child [11]. These actions are likely to help minimize regrets that the parent may feel after their child’s death. Nevertheless, in the context of caring for a child with a life-limiting condition, it is not uncommon for parents to experience several occasions when they believe their child’s death to be imminent, thus making it difficult for them to know when it is really time to say a final goodbye.

It is important to acknowledge differences in parental bereavement processes following the death of a child due to cancer, relative to the death of a child due to a non-cancer, life-limiting illness. In the context of many life-limiting, non-cancer conditions, parents typically learn at the time of diagnosis that, although there may be uncertainty regarding how long their child is likely to live, they as parents are likely to outlive their child. This information is likely to gradually reshape their cognitive schemas. In contrast, parents whose child has been diagnosed with cancer, may recognize the possibility of their child’s death, however, they typically retain hope that their child will make a full recovery and enjoy a full life expectancy. These parents are therefore more inclined to cling to their existing schemas for as long as possible, hoping that the treatments will enable a return to health and normality.

## 3. The Role of Risk and Resilience Factors in Parental Bereavement

Over the past decade the Integrative Risk Factor Framework of Bereavement [3] has been valuable in drawing attention to a broad array of risk factors that may adversely impact on parental bereavement. This framework grouped potential risk factors as: (i) loss-oriented and restoration-oriented stressors; (ii) intra-personal factors, which are stable factors that are intrinsic to the bereaved individual; (iii) inter-personal factors, which are stable factors external to the individual (e.g., social support and culture); and (iv) coping and appraisal. These factors, alone and in combination, have the potential to impact on the ability of bereaved parents to resume functioning with necessary and valued activities. However, advances in the resilience literature suggest that intra-personal factors and inter-personal factors should not only be considered as potential risk factors for poorer bereavement outcomes, but that they should also be considered as possible resilience factors [12,13]. Although a risk-focussed approach has been useful in some fields of medicine, such as investigating infectious diseases, when investigating more complex conditions with biopsychosocial components, a more comprehensive consideration of both risk and resilience factors is likely to be beneficial [14].

Resilience may be regarded as an individual’s ability to respond effectively to challenges or adversity. In some cases, resilience factors may be the opposite end of the spectrum of risk factors, for example, good marital communication may be considered a resilience factor, whereas poor marital communication may be a risk factor. However, risk and resilience factors are not always at opposite ends of the same continuum. For example, substance abuse confers a risk, but it cannot be said that its absence confers any protective value.

Some definitions of resilience highlight an individual’s sustainability of purpose in the face of stress [13]. In recent years, the concept of resilience has been applied to the health psychology literature to identify why some individuals adjust to chronic health stressors more readily than others [15,16]. Resilience has been identified as being of importance in the bereavement literature, with the potential to help account for why some individuals are able to resume functioning more readily than others, despite experiencing painful and life-changing losses [17,18]. From a theoretical perspective, resilience factors may operate in a number of ways. Firstly, they may buffer or serve to compensate or minimize the effect of the stressor or loss in some way. For example, having other children in the family may prevent a parent from facing a childless existence, thus enabling, indeed requiring, them to maintain their role as parent. Secondly, resilience factors may facilitate the individual’s process of recovery. In this case, the stressor or loss may be experienced just as acutely, but the resilience mechanisms may facilitate coping and accelerate the process of recovery [19,20]. For example, good marital communication may enable the bereaved parents to assist each other with more effective problem-solving.

**Figure 1:**
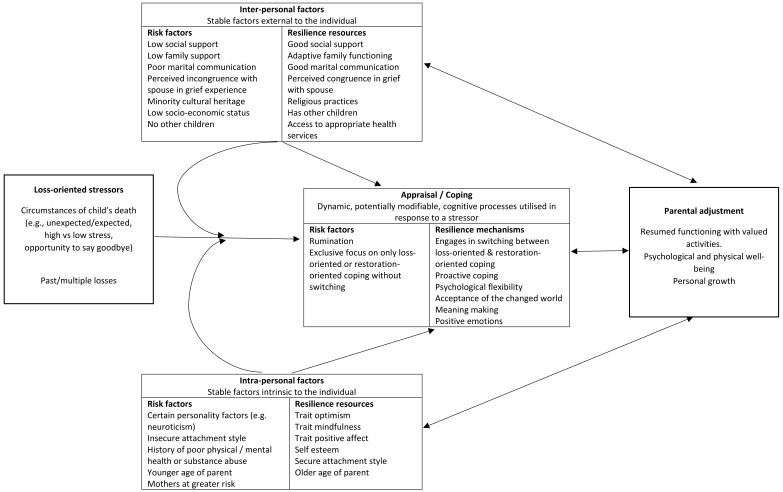
Risk and Resilience Model of Parental Bereavement.

The current paper draws from the multi-faceted risk framework of bereavement outlined by Stroebe et al. [3] and integrates this framework with a more comprehensive consideration of resilience factors. Thus, we have proposed a new model, namely the Risk and Resilience Model of Parental Bereavement (see Figure 1). Like the Stroebe et al. [3] framework of risk factors, this model groups loss-oriented factors, intra-personal factors, inter-personal factors, and appraisal and coping, separately. However, each of these classes of factors is considered in terms of potential risk and resilience influences on parental bereavement outcomes. This more comprehensive and holistic framework for considering how multiple risk and resilience factors interact is paramount to an improved understanding of parental bereavement outcomes, promoting theoretically-driven research, and guiding evidence-based clinical practice. The specific factors outlined in Figure 1 have been included based on available empirical or theoretical justification. Following a brief discussion of the varied nature of parental bereavement outcomes, the literature on each of the four classes of bereavement risk and resilience factors will be reviewed, where possible with a particular focus on parental bereavement associated with the death of a child following a life-limiting condition.

## 4. Parental Bereavement Outcomes

Whilst there is consensus that grief is a normal experience following a major loss, it is difficult to define the process of normal grief. It is generally recognized that the grief response is dynamic, pervasive and highly individualized [21]. The process of grief is not linear and does not fit neatly into predetermined categories. The death of a child commonly results in detrimental effects on the psychological and physical well-being of parents [22,23]. Psychological responses to parental bereavement may include heightened anxiety, depression, suicidal ideation, and reduced quality of life [22,24,25,26]. Increased risk of psychiatric hospitalization has also been reported, especially in mothers [27]. Detrimental physical outcomes that have been reported in response to parental bereavement include a greater risk of cardiovascular problems [28], cancer [26], and higher rates of mortality due to natural and unnatural causes [26]. A wide range of detrimental social [29], marital [30], occupational and financial consequences [31] have also been reported amongst bereaved parents.

The adverse outcomes listed above are certainly not experienced by all individuals, with there being considerable variability in symptoms experienced. Moreover, there is also much variability in the duration of intense grief reactions [22]. Most bereaved individuals return to relatively normal functioning, as judged by external standards, within a relatively short time-frame [1], even if their experience of life is now different.

Over the years, various terms and classifications have been used to describe intense and debilitating grief reactions, including persistent complex bereavement disorder, prolonged grief disorder, bereavement-related major depression, complicated grief, pathological grief. Although there remain differences in opinion as to how best to classify these individuals [32,33], it is generally recognized that 5–10% of bereaved individuals experience significant and prolonged impairment of functioning [34]. It is not clear what these figures are for parental bereavement following the loss of a child due to a life-limiting condition, however it has been found that the context of an expected death poses lower risk than unexpected deaths [35]. Where data are available, the current paper will consider risk and resilience factors associated with such intense and debilitating grief reactions; however, the paper will primarily encompass discussion of risk and resilience factors associated with the full spectrum of grief reactions.

## 5. Loss-Oriented Stressors

### 5.1. Circumstances Surrounding the Death

Once parents recognize that their child has a life-limiting condition and may die in the foreseeable future, they may begin to consider and plan for the circumstances surrounding their child’s impending death. Palliative care teams often discuss with parents issues such as: (i) whether they have a preference for where the child dies (e.g., at home or at hospital); (ii) who they would like to be present; (iii) what medical interventions are to be used and when these should be ceased; (iv) what will happen to the body immediately after the death; and (v) what psychological support is available to the family regarding this decision-making. Many of the above choices are not always open to the parents, but often there is some scope for parental preferences. It is generally assumed by clinicians that the parents’ choices should be respected wherever possible.

Although there is some evidence that different causes of death (illness versus injury/accident) may be associated with different parental bereavement outcomes [7,8], there is little evidence on whether specific circumstances surrounding the death are associated with more favourable bereavement outcomes than others. A study by Grande et al. [36] considered whether the location of the child’s death was associated with parental bereavement outcomes six weeks and six months later. They found that parents had better outcomes six weeks following a home death rather than a hospital death, but that there was no difference at six months. Another study also found that fathers reported higher levels of depression, anxiety and stress when their child with cancer died in hospital rather than home [37]. However, given the complex nature of circumstances, and that location of death was not a matter of chance, one cannot infer causality from such associations. For example, the above results may have been due to families who were not coping well in the lead up to the death, being more likely to choose a hospital-based, rather than home-based, death. It should also be noted that another study found that the circumstances associated with a child’s death were found to have a lesser impact on parental bereavement outcomes than parental coping styles [38].

Anecdotally, parents report valuing the opportunity to say a final goodbye to their child [11]. This is more likely to be possible when there is a recognition that the child’s death is imminent, enabling parents to participate in tasks, processes and rituals that they deemed important prior to their child’s death [11].

### 5.2. Multiple Losses

The way in which parents have responded to, and coped with, previous losses may give some indication of how they are likely to respond to an impending death of a child with a life-limiting condition. Parents who have had a child die from a life-limiting genetic disorder may have another child with the same genetic disorder, which may result in more than one grief experience. It may be clinically useful to ask parents about previous losses, as it can provide useful information about their ability to access and utilize their intra-personal and inter-personal resources when faced with bereavement. Moreover, it is possible that a history of multiple losses may also render individuals more vulnerable to poorer adjustment outcomes. There are relatively few data about the effects of multiple losses and previous grief experiences on the subsequent experience of loss. A study with 190 adult participants found that a history of more than two losses was associated with a higher probability of developing complicated grief, with the number of losses having a cumulative effect [39]. In contrast, another study with gay men found no association between number of losses and grief intensity [40]. Simply considering the number of losses may not be as informative as considering other factors associated with earlier losses, such as coping style and social support. The time period between bereavement experiences may also be a relevant factor, with suggestions that if a subsequent loss occurs soon after an earlier loss, it can “interrupt” a normal bereavement process, leading to poorer adjustment [39,41].

Bereaved individuals experiencing other concurrent losses, such as loss of employment, divorce, and significant financial setbacks, are likely to be at greater risk of poor bereavement outcomes [42,43]. Their coping resources may already be stretched to capacity prior to the bereavement, thus leaving them with few remaining adaptive resources. Moreover, concurrent stressors, such as marital tension or work difficulties, are likely to continue into the bereavement phase, and may therefore exceed the parent’s coping resources making it more difficult for them to transition into the new reality of their environment.

## 6. Inter-Personal Resources

Inter-personal resources are those that originate within the social or environmental context within which the bereaved parent is functioning and include things like marital factors, social support and religious practices.

### 6.1. Marital Factors

Not only do spouses need to grapple with their own loss following the death of a child, they also need to cope with their partner’s grief reaction [44]. Spouses commonly experience and respond to the death of a child differently, which, in the absence of good communication, may result in marital tension. Differences may occur in any number of areas including: patterns of continuing bonds with the deceased child [45], willingness to express emotions [46], timing of readiness to resume usual roles and activities, and readiness to resume sexual intimacy [47]. A recent study of 229 bereaved couples found that bereaved parents who perceived that they had dissimilar levels of grief to their spouse (less or more grief), whilst controlling for actual differences in grief, reported lower relationship satisfaction, compared with bereaved parents who perceived more similar levels of grief [48]. Moreover, the negative effects of the perceived dissimilarity were found to increase over time. Teaching couples about sources of incongruence in their grief has been suggested as an intervention to enhance marital cohesion and relationship quality [49]. Good communication between spouses may serve as a protective buffer to minimize the impact of spousal differences in their grief. Notably, when assessed within the first two years of the loss of their infant, bereaved wives who perceived a lack of opportunity to share their thoughts and feelings with their husbands were found to experience more intense grief reactions two years later than wives who reported more opportunity to share thoughts and feelings with their husbands [50].

In light of the stress that individuals and couples experience following the death of a child, marital disruption is a common, though not inevitable, consequence [51]. Pre-existing marital difficulties, especially following the prolonged illness of a child, may manifest in more overt discord following the death of a child. A study comparing the divorce rates of bereaved parents with a control group of non-bereaved parents found somewhat higher divorce rates among bereaved parents in the first 6 months following the death (5–6%), relative to non-bereaved parents (0.5–1.5%) [30]. However, marital discord is not inevitable following the death of a child, and in some cases parental relationships may even strengthen following the death of a child [51].

It has been suggested that marital disruption may be greater if the bereavement occurs early in married life relative to mid-to-later life [7]. Consistent with this premise, bereavement following neonatal death has been associated with particularly high levels of subsequent marital disruption [30]. Marital disruption has been found to be less if there are other children in the family at the time of the death [28], presumably giving the parents a unified purpose in caring for their other child/children.

### 6.2. Social Support

Social support, particularly an individual’s perception of social support [52], is recognized as being beneficial to all individuals, irrespective of whether or not they currently face bereavement [20]. Nevertheless, social support warrants particular attention in the context of bereavement. Social networks are known to alter in the context of caring for someone with a long-term illness. While some individuals report that their social network galvanized around them to provide support and care, others describe increasing social isolation [53,54], particularly if their child had a long illness. It is well accepted that individuals and families who report having good social support cope with stressors more effectively [55]. These findings have also been found to hold true within the context of bereavement following the death of a child [4].

This leads to the question about whether social support-based interventions are able to improve bereavement outcomes. Social support interventions have been developed across a broad range of contexts, and have differed widely in terms of efficacy (for a review see Hogan et al.) [56]. Despite general acceptance of the importance of social support when faced with bereavement, there has been relatively little research investigating the efficacy of social support interventions in the bereavement context, with the available evidence being mixed. Support groups in the context of parental bereavement have been frequently recommended [57,58], however, the evidence base for such interventions remains relatively weak. An extensive review of the literature [20] found there to be limited evidence to support the widely held view that social support serves as a buffer against the impact of bereavement or facilitates recovery. Many of the studies to report positive effects of social support intervention have either had low numbers or utilized a qualitative research design [59].

More recently, however, a noteworthy randomized control trial [60], albeit using block randomization according to the hospital site at which the child died, with 103 bereaved Finnish fathers, evaluated a social support intervention for bereaved fathers. The intervention consisted of an information support package, peer contact, and health care personnel contact, aimed at showing compassion and care for fathers and providing concrete aid. When assessed six months post-bereavement, fathers in the intervention condition (*n* = 62) reported stronger personal growth and some lower grief reaction scores (e.g., blame and anger) relative to fathers in the control condition (*n* = 41). A similar study, with essentially the same intervention program, was carried out with 136 bereaved Finnish mothers [61]. Although mothers who reported greater perceived social support also reported lower grief reactions, there were no significant differences in maternal grief reactions between mothers in the intervention condition (*n* = 83) and those in the control condition (*n* = 53).

A cross-sectional study with bereaved mothers found that mothers who endorsed having attended a bereavement support group reported significantly fewer traumatic stress symptoms than women who did not participate [62]. However, the women were not randomly allocated to whether they participated in a support group or not, and therefore other factors may account for these results—for example, the support group participants may have been engaging in more active coping strategies in the first place prompting them to join the support groups.

A Swedish population-based study with 449 parents who had lost a child to cancer 4–9 years earlier found that parents who reported having access to “psychological support” in the last month of the child’s life, were more likely to report that they had worked through their grief at the time of the assessment [63]. However, given the retrospective nature of the study, the possible role of memory bias should be acknowledged. Individuals coping better at the time of the assessment may have had a more favourable recall of the psychological support available to them in the month prior to their child’s death.

Not all bereaved parents are likely to express the same desire for social support groups. For example, there is some suggestion that social support groups are more desired by parents who did not have adequate forewarning about their child’s death [64]. One study found that 80% of bereaved parents who opted to participate in a support group had lost their child without adequate forewarning. In contrast, 76% of parents who opted not to participate in a support group had lost their child after a period of anticipatory grief [64]. Gender differences may also impact on social support group preferences. Males have been found to be less likely to seek emotional support than females, and to be more likely to seek instrumental support rather than emotional support [65]. Moreover, one study found that fathers may be more likely to participate in electronic support groups than face-to-face support groups [66], which is an area that may warrant future research in the bereavement context.

The considerable variability in the efficacy of social support interventions with bereaved parents is, in part, likely to be related to the very varied nature of social support interventions. Some such interventions aim to provide support directly, whereas others aim to establish skills or make changes that are likely to enhance naturally occurring social support. Some interventions offer social support from peers (e.g., family, friends, or other individuals in a similar situation) whereas other interventions offer support from a health professional, some may be parent-driven discussions and others highly structured groups led by a health professional. Moreover, intervention formats may differ in terms of whether they are individually-based versus group-based, or whether they are face-to-face versus electronic. More research is needed into the possibility of matching types of social support interventions to the individual.

Acknowledging some exceptions (as listed above), much of the research investigating social support in the bereavement context has focused on marital bereavement [20]. It is important to recognize some important differences in these contexts. The losses commonly associated with marital bereavement, such as loss of instrumental, emotional and validational support [67], may be partially compensated by effective social support from family and friends [20]. However, the losses associated with the death of a child are somewhat less tangible, and arguably more difficult for family and friends to compensate for in any significant way.

Anecdotally, migrant families commonly report longing for the support of their parents, but may be practically or financially unable to make the necessary travel arrangements.

Cultural heritage may shape and define the way in which individuals express their grief [68,69], such as “culturally approved” somatization [70]. Consequently, first generation immigrants may experience a more challenging bereavement experience if they are cut off from their traditional cultural groups, but have not assimilated into adopting the grief cultural practices of the society in which they now reside [71,72]. The published scientific bereavement literature is very much from a western cultural perspective, leaving health professionals reliant on whatever training is available to them about immigrant cultural practices.

### 6.3. Religious Practices

The literature on whether parental religious practices are related to bereavement outcomes is mixed. There is some literature on the benefits of religion when coping with stressful life events [73,74]. Walsh et al. [74] found that individuals who professed stronger spiritual beliefs of any religious persuasion (and distinct from religious observance) seemed to have better and more rapid resolution of their grief than individuals who reported no spiritual beliefs. Other researchers have found variable, and at times worse, adjustment among the more religious in a bereavement context [75]. One study found that, when 102 newly bereaved individuals were assessed, frequent church attenders responded with higher optimism and social desirability, but more repression of grief responses than less frequent church attenders [76].

## 7. Intra-Personal Resources

Intra-personal resources refer to characteristics that are stable and intrinsic to the bereaved individual (e.g., personality, attachment style, gender, predisposing personal vulnerabilities such as substance abuse, physical or mental health problems).

### 7.1. Personality

Personality variables are stable intrapersonal constructs that are predictive of behavioural responses across both time and situations, and are therefore likely to impact how individuals respond to adverse life events, such as bereavement. They may serve as either risk or resilience factors. Personality variables may impact bereavement outcomes due to their influence on an individual’s approach to coping. Some personality variables have been found to be associated with less adaptive approaches to coping, poorer inter-personal interactions, and poorer adjustment to stressors. Other personality variables have been found to be associated with greater resilience in the face of life stressors. Within the bereavement context, neuroticism has been consistently identified as a significant risk factor of poorer outcomes [77], whereas possible personality resilience factors include trait optimism [78,79], psychological flexibility [80], and trait mindfulness [81]. The literature on these factors will be reviewed.

Neuroticism may be defined as the tendency to respond to threat, frustration or loss with negative emotions. Hence it is not surprising that elevated scores on measures of neuroticism have been found to be associated with poorer bereavement outcomes [77]. This may be due the coping responses that individuals high in neuroticism engage in. In a study of 325 bereaved individuals, individuals scoring higher on a measure of neuroticism were found to be less likely to engage in strategies that contribute to meaning-making, or finding understanding in the situation [82]. Another study found that individuals who were high in neuroticism provided narratives about their loss that were more self-focused [83]. The investigators suggested that this served the purpose of obtaining emotional validation and was reminiscent of ruminative coping.

It is important, however, to acknowledge the overlapping conceptual nature of poorer bereavement outcomes, such as elevated anxiety and distress, and behavioural characteristics commonly found in individuals high in neuroticism [77,84]. Similarly, individuals who are high in extraversion are more likely to demonstrate a tendency to be more sociable, active and assertive, characteristics typically associated with better behavioural outcomes. These characteristic similarities may make it difficult to accurately determine the impact of personality variables on measured outcomes, particularly as measures of personality prior to bereavement are often not available.

As growing attention in the literature is being devoted to factors that may make some individuals more resilient in the face of adverse life events [15,17], consideration should be given to which personality factors are likely to be associated with better adjustment following parental bereavement. Trait optimism has been defined as the tendency to adopt favourable expectancies for the future [85]. In the context of parental bereavement, this may manifest as the parent’s capacity to envisage engaging in valued activities again in the future, in a world altered by the death of their child. Although depression scores in the clinical range are common in bereavement, trait optimism has been found to predict a shorter duration of elevated depression scores [79]. Moreover, individuals with high trait optimism one month post-bereavement have been found to have significantly better psychological adjustment at 6, 12 and 18 months post-bereavement relative to pessimists [86], even when controlling for differential coping strategies.

Trait optimism is a relatively stable individual difference variable that reflects a predisposition to expect favourable expectancies for one’s future [87]. It has been shown to be associated with favourable adjustment outcomes across a wide range of contexts, including surgery, cardiovascular disease, respiratory failure, in vitro fertilization, cancer, AIDS progression, caregiving, and academic examinations (for reviews see [87,88,89]). One can speculate numerous possible mechanisms by which trait optimism can lead to more favourable outcomes: (i) optimism has been found to be associated with greater approach-based coping responses and fewer avoidance-based coping [89]; (ii) optimism is generally regarded as socially desirable, and may therefore be associated with the availability of greater social support [90]; (iii) optimists are more confident about eventually attaining desired outcomes, which in the context of bereavement may be the ability to re-engage in valued activities, and are therefore more likely to keep trying in the face of difficulties encountered [87]; (iv) optimism is by definition the opposite of hopelessness, the latter being associated with depression and poorer psychological adjustment [91]; and (v) optimism is associated with positive affect, which is likely to confer direct benefits across a range of contexts [92]. Although most bereaved parents would find it difficult to recognize any feelings of optimism following their child’s death, and may find the term difficult or even offensive, there is likely to be considerable variability with regard to their ability to conceive of engaging in valued activities again some time in the future. To date there have been relatively few studies investigating the relationship between trait optimism and bereavement outcomes. Nevertheless, a longitudinal study with individuals in the first year after their loss, and then 6 months, and 15 months later, found that trait optimism was inversely associated with concurrent and future levels of depression and prolonged grief [78].

Although psychological flexibility is not a new concept, interest in its application as a personality resilience factor in the health psychology context has emerged relatively recently [80,93]. The term psychological flexibility (also sometimes referred to as regulatory control, executive control or response modulation) has been used to refer to an individual’s capacity to efficiently regulate one’s behaviour, emotions and coping, based on an awareness of contextual demands, repertoire of coping skills, and responsivity to internal and external feedback [80]. Psychological flexibility has been consistently found to be associated with better overall health and adjustment [94]. Within the context of bereavement, psychological flexibility is likely to have a particularly important role. The dual-process theory of bereavement highlights the importance of shifting attention between a loss-oriented focus (namely a focus on appraising and processing some aspect of the loss experience) and a restoration-oriented focus (namely a focus on reorienting oneself in a changed world without the deceased person) [95]. Arguably, individuals with a greater capacity for psychological flexibility may be able to engage in this shifting more effectively. There has been little empirical investigation into the concept of psychological flexibility and bereavement. A related concept of emotional expressive flexibility has been examined in the context of bereaved spouses (a subset of whom had complicated grief) and non-bereaved married adults [96]. Adults experiencing complicated grief were found to display deficits in expressive flexibility relative to asymptomatic bereaved adults and married controls. In the absence of longitudinal studies it is not possible to infer any causal direction.

Trait mindfulness is the tendency to purposefully and non-judgmentally attend to the present moment. It has been found to be associated with psychological well-being across a range of domains [97]. Within the bereavement context, greater mindfulness may enable individuals to allow themselves to experience the many emotions of grief in a non-judgmental way. Clinical approaches have been developed to facilitate greater mindfulness in bereaved individuals [81,98], though few data have thus far been reported on the efficacy of such approaches.

### 7.2. Attachment Style

An individual’s attachment style influences their behavioural, emotional and cognitive patterns of responding, impacting on their appraisal and coping with life stressors. The theoretical and empirical literature regarding the relationship between parental attachment variables and the paediatric palliative care context has been comprehensively reviewed by Kearney and Byrne [99]. Notably insecure attachment styles (e.g., anxious or avoidant patterns) have been found to be associated with decreased resilience, complicated grief reactions, poorer psychological outcomes, and marital distress [44,84,100,101,102,103]. In contrast, secure parental attachment styles have been found to be associated with more effective distress management, active support seeking, better engagement with healthcare providers, and better coping responses [99,104,105], which are likely to contribute to better bereavement outcomes [99]. Notably, there has been little empirical work investigating potential differences in the attachment style of parents of a child with a life-limiting condition, relative to the attachment style of parents of a child with normal life expectancy.

In addition to considering established parental attachment styles, attachment theory holds that bereavement outcomes are also contingent on an individual revising their internal working model of attachment to the deceased individual in accord with the changes in the external world brought about by the loss [106]. Failure to make such revision is what prominent attachment theorists such as Bowlby [107] consider to be at the heart of complicated grief.

### 7.3. Gender

Gender differences in relation to bereavement outcomes have mostly been considered in the context of the death of a spouse, with greater rates of depression and mortality documented in men relative to women, and gender differences in coping responses (for reviews see [23,108]). However, the issues related to bereavement following the death of child relative to the death of a spouse differ in a number of key ways, making it difficult to generalize across contexts. Research examining parental bereavement typically considers a younger cohort of bereaved individuals, with most fathers and some mothers likely to be employed. Bereaved parents are also more likely to have other family members living in the household, such as their spouse and/or other children for whom they continue to be responsible.

Mothers have been found to rate their grief feelings higher than fathers following the death of an infant [109]. It is not clear, though, whether women experience these reactions more intensely or whether men experience bereavement in a way that is not measured by the assessment instruments commonly used.

A number of specific variables have been identified that differ for bereaved men and women that may impact on bereavement outcomes. Women tend to talk more about their feelings to others, while men tend to minimize the expression of painful emotions and cope with negative emotions in a more solitary way [46], immersing themselves in practical tasks and in their work [110]. Women commonly have additional confidants outside their marriage, whereas men are more likely to rely exclusively on their wives as confidants [108]. Discordant coping styles between husbands and wives are likely to add to the stress of each individual, with feelings that their spouse does not understand them.

### 7.4. Predisposing Personal Vulnerabilities

There are numerous pre-existing intrapersonal factors, such as substance abuse, mental health problems, and poor physical health, which are widely recognized to compromise an individual’s coping resources and place them at greater risk of poor bereavement outcomes [43,111]. Excessive use of alcohol has been widely documented among bereaved individuals, especially males [112]. The use of alcohol as a maladaptive avoidance strategy during the stressful period of caring for a child with a life-limiting condition is likely to place the parent at significant risk of substance abuse problems in the bereavement period. Unfortunately, only a relatively small proportion of the bereavement literature has utilized information collected from carers before the death of a spouse or child, making it difficult to gain a clear understanding of predisposing factors.

Psychological problems are relatively more common among long-term carers than in the general population [113], largely related to the high levels of stress which they experience. The intense stress associated with bereavement is likely to further exacerbate any pre-existing psychological or physical conditions [43]. An early study found that 60 per cent of individuals who committed suicide following bereavement had undergone psychiatric treatment prior to bereavement [114].

Pre-existing physical problems may be more common prior to bereavement following the death of a spouse than the death of a child, given that the loss of a spouse generally occurs at an older age. Nevertheless, anecdotally, parents caring for a seriously unwell child over an extended period of time commonly overlook their own physical healthcare needs, and therefore place their own health at greater risk. Any physical problems may be further exacerbated when faced with the intense stress of bereavement.

Despite the paucity of research to date which has utilized information collected from carers before the death of a child, such research is more feasible in the context of caring for a child with a life-limiting condition. In this context, clinicians (and researchers) may have contact with parents in the weeks and months prior to a child’s death. At a minimum, this should allow for a thorough investigation of possible predisposing risk factors, and ideally may provide the opportunity for appropriate supports and interventions to be implemented to address the relevant risk factors.

## 8. Coping and Appraisal

Coping may broadly be considered as the process by which an individual appraises the personal significance of a situation or event and the options that they have for responding to that situation or event [3]. Over the years, individuals typically develop a tendency to utilize certain coping styles. However, when faced with specific challenges, the individual must appraise the situation and their capacity to respond to it, and apply particular coping strategies accordingly.

Coping is known to be associated with, and likely to mediate, the relationship between interpersonal and intrapersonal variables with adjustment [3,77,115]. Within the context of bereavement, the coping process is of particular importance because it offers possible targets for effective intervention, given that these constructs may be amenable to change [3,116]. In an early paper addressing intervention strategies for coping with transitions, a useful list of coping competencies was outlined [117]. These coping competencies included skills for assessing, developing and utilizing internal and external resources, skills for managing emotional and physiological distress, and skills associated with planning and implementing change. Despite these transition-based coping competencies being articulated more than 35 years ago, more research is needed to investigate the role of coping interventions in the context of parental bereavement.

The majority of the literature on coping pertains to situations that have occurred and present a current challenge or threat to the individual. However, a small body of literature addresses what has been referred to as proactive coping, pertaining to anticipated threats or stressors [118]. The literature regarding bereavement following the death of a child due to a life-limiting condition would benefit from a consideration of both types of coping, given that parents face many ongoing stressors whilst caring for a child with a life-limiting condition, whilst also being mindful that they will one day need to face their child’s death. In other contexts, proactive coping has been found to be beneficial, and indeed teachable, heightening an individual’s awareness of their personal and social resources, so that they are better placed to make effective coping decisions [119]. However, the concept of proactive coping has, to date, not been well applied to parental bereavement following a child’s death due to a life-limiting condition, and warrants further research.

It is generally recognized that specific coping strategies are not uniformly effective (or ineffective) across all contexts and situations. Instead, an individual’s coping efficacy is dependent on an efficient process of self-regulation, which requires an awareness of contextual demands, availability of a range of coping skills to select from, and responsivity to internal and external feedback [80]. A small-scale study investigating the coping of bereaved parents found that the coping strategies used by those bereaved for 18 months or less differed considerably from non-bereaved normative samples, whereas those bereaved for more than 18 months engaged in coping strategies similar to normative samples [120]. The efficacy of the coping strategies, however, was not examined.

Within the bereavement literature, Stroebe and Schut [95] made a distinction between loss-oriented coping (namely a focus on appraising and processing some aspect of the loss experience) and restoration-oriented coping (namely a focus on reorienting oneself in a changed world without the deceased person). The dual process theory of bereavement holds that oscillation between these different types of cognitive processing is essential for adaptive bereavement outcomes and that over time more focus is placed on a restoration orientation and less on a loss orientation [121,122]. In a study of 219 couples following the death of their child, Wijngaards-de Meij et al. investigated parental use of restoration-coping and loss-oriented coping [123]. Although utilizing quite a limited set of items to assess coping, Wijngaards-de Meij et al. found that a greater focus on future-oriented, restoration-coping, irrespective of the amount of loss-oriented coping that was used, was associated with more beneficial outcomes [123]. Moreover, when women made greater use of restoration coping, their husbands also benefited, showing lower levels of depression.

Although psychological flexibility is sometimes regarded as a personality dimension, it may also be considered as a more malleable, cognitive coping process. Within the Acceptance and Commitment Therapy (ACT) framework, psychological flexibility has been taken to refer to an ability to be present-focussed, acting in a manner consistent with one’s values, even in the presence of interfering thoughts and emotions [93]. ACT interventions with the parents of children with a life-limiting condition have successfully increased aspects of parental psychological flexibility [124]. However, to our knowledge, studies have not investigated parental psychological flexibility following the death of a child following a life-limiting condition.

Similarly, within the body of literature on mindfulness, the concept of acceptance has emerged as being of importance in the face of life stressors [125]. Within the trauma literature, acceptance has been found to be associated with greater psychological adjustment following exposure to trauma (for review, see [125]). Within the context of bereavement, the concept of acceptance has been integral to clinical mindfulness interventions that have been developed, such as based on the ATTEND (attunement, trust, touch, egalitarianism, nuance, and death education) framework [81]. However, evaluations of these interventions have thus far been limited [126].

Meaning-making is a term that has emerged and proliferated in the literature over the past decade, referring to the restoration of meaning following a highly stressful situation [127]. Meaning-making requires the integration of the meaning given to a stressful event with one’s global orienting system or cognitive framework [127]. Highly stressful situations have the potential to challenge one’s global cognitive systems. The extent to which the meaning that an individual attributes to a stressful event is discrepant with their global cognitive system is likely to determine the extent to which they experience distress. It has been found that the degree to which parents have made sense or meaning out of their child’s unexpected death was inversely related to their degree of distress [128], albeit one study found this relationship was only significant in the first year of bereavement [129].

The nature of a child’s death has been found to impact on the ability of parents to find meaning in the situation. Parents whose child died a violent death (i.e., accident, homicide or suicide) found it more challenging to make-meaning of the situation relative to parents whose child died a non-violent death (perinatal, natural anticipated, or natural sudden) [130]. There has been little work investigating the process of meaning-making specifically among parents of children who died from a life-limiting disorder.

Positive emotions have been suggested to serve a restorative role in bereavement, and as a catalyst for meaning finding and benefit finding [92]. Moskowitz, Folkman and Acree [79] noted a positive association between positive affect and bereavement outcomes, which they attributed to an increased likelihood to engage in positive reappraisal. Moreover, a positive affect renders individuals more able to seek social support. However, the overlapping nature of measures of positive emotions and of bereavement outcomes makes it difficult to disentangle these constructs or to consider issues of causality.

Rumination is a coping style characterized by recurrent, self-focused negative thinking. It is widely regarded as a normal part of grieving. However, more extreme rumination is likely to be problematic and a predictor of poorer bereavement outcomes [131]. In a study with 55 bereaved individuals (following the death of a first degree relative within the previous three years), greater rumination was associated with symptoms of psychopathology over a 12-month period [132]. It has been suggested that the repeated focus of attention on negative emotions associated with rumination interferes with problem solving capabilities, and impedes instrumental behaviour and the utilization of social support [131]. Females and individuals with lower social support have been found to engage in more ruminating behaviour following the death of a loved one [116].

Rumination used to be considered a confrontational strategy, requiring individuals to confront and experience distress and negative emotions. More recently, though, rumination has been appraised as an avoidance strategy [133], whereby an individual focuses disproportionately on loss-oriented coping with little attention to restoration-oriented coping. According to this view, individuals engage in ruminative thinking, dwelling on negative aspects of their personal loss, and in so doing avoid confronting the new realities of their life and fail to restructure their cognitive schemata accordingly.

The nature of the cognitive appraisal that an individual engages in may be influenced by their cultural beliefs. For example, individuals holding traditional Chinese beliefs are more likely than individuals holding Western beliefs to adopt an external locus of control and attribute the cause of a death to predestined rules or higher powers such as sick *qi* (negative energy that could pass to the unlucky) or evil spirits [134]. This remains an under-researched area.

## 9. Challenges to Carrying Out Bereavement Research

Studying the bereavement process and factors associated with parental bereavement outcomes is fraught with challenges [135]. Key areas of difficulty relate to participant recruitment, inter-relatedness of variables, and the selection and use of multiple outcome measures. Each of these areas of difficulty will be briefly described.

Research participation is voluntary; hence self-selection is likely to influence who participates in bereavement research [136]. Individuals may decline to participate due to: depressed mood, feeling they are too upset to answer questions about their bereavement, fear that participation may increase their grief, or greater use of avoidant coping strategies. It is therefore possible that individuals who are most disabled by grief, perhaps meeting the Diagnostic and Statistical Manual of Mental Disorders—5th Edition (DSM-5) criteria of persistent complex bereavement disorder, may commonly not participate in bereavement research. Individuals who agree to participate in bereavement research may be more willing to talk about their experience, and therefore may already be engaging in more adaptive coping strategies. Notably, given that women are more likely to seek social support and talk about their feelings and experiences [46], they may be more likely to engage in bereavement research. This issue is likely to be magnified if recruitment occurs through bereavement support services, which may predominantly be utilized by individuals who are willing to talk about their experiences. Alternatively, it is conceivable that individuals who are more distressed may be more likely to take up the opportunity to talk with someone about their feelings [136]. Moreover, there is evidence of a selective invitation bias in paediatric palliative care research, whereby not all eligible families are invited to participate due to non-random factors [137].

Factors that potentially render parents at greater or lower risk of poor bereavement outcomes are often difficult to disentangle from complex, inter-related circumstances and attitudes. For example, decisions regarding the preferred location of the child’s death may be related to the family’s coping efficacy, their perceptions of how well their child’s symptoms are able to be managed outside of hospital, the supports and services available to the family, and considerations regarding the presence of other siblings. Randomization is rarely appropriate to study such factors in a methodologically rigorous way. A multivariate statistical approach would also be useful in minimizing the reporting of spurious associations, but requires an adequate sample size.

When carrying out research with bereaved individuals it is difficult to find the right balance between assessing multiple outcome domains of possible interest and not wanting to over-burden bereaved parents. Many studies have considered only a single measure of bereavement outcome, failing to acknowledge the complex and varied ways in which different individuals respond to the death of a child. For example, it is increasingly recognized that mothers and fathers experience the loss of a child differently [108]. Some outcome measures may be more sensitive to identifying the responses of women rather than men, or vice versa.

## 10. Future Directions for Research and Clinical Practice

It is important to achieve better alignment between quality research in the area of parental bereavement, particularly in the context of a death following life-limiting condition, and clinical practice. Although the natural trajectory of bereavement has been documented in the context of bereaved older spouses [138], at present the natural history of bereavement in parents following the death of a child has not been well studied. Prospective studies in this area are needed, though challenging due to the relatively small numbers of children known to be approaching death and the difficulty of engaging parents at this time. Multi-centre collaboration would be useful to achieve sufficient sample sizes. Alternatively, the use of large-scale, longitudinal databases (i.e., Big Data) would not only provide useful longitudinal data, but, importantly, also avoid many self-selection and recruitment biases common in this area of research, given that participation is not specific to their bereavement status.

The evidence for clinical interventions with bereaved parents is currently poor [139]. In a systematic review by Endo et al. [139], nine articles were retrieved, describing eight randomized controlled trials of clinical interventions with bereaved parents or siblings following a child’s death. The interventions were varied, and included support groups, counselling, psychotherapy and crisis intervention. However, the authors of the systematic review concluded that there was limited evidence of sufficient quality to support the intervention techniques used. Similarly, the literature in other areas of bereavement suggests that most individuals regain their pre-loss levels of functioning after a transitory period of distress (e.g., 6–12 months) irrespective of whether they receive any intervention [140]. The authors of an earlier meta-analysis evaluating the efficacy of psychotherapeutic interventions for bereavement concluded that more favourable outcomes were obtained for programs that specifically targeted bereaved individuals experiencing most marked difficulties [141].

Within the context of the Risk and Resilience Model of Parental Bereavement proposed in this paper, the current review has identified various inter-personal and intra-personal factors that may positively or detrimentally impact parental bereavement outcomes. These risk and resilience factors may be identified in the weeks, months, or even years prior to a child’s death within the supportive context of a relationship with a palliative healthcare provider. Many risk factors, such as low social support, previous losses, predisposing personal vulnerabilities (such as psychiatric history or history of substance abuse), are likely to be identifiable through clinical interview. Other factors, such as attachment style, trait mindfulness and psychological flexibility, may warrant the use of brief, validated questionnaires. A clearer identification of which parents are at greatest risk of adverse bereavement outcomes will help pave the way for the development and evaluation of targeted interventions. Consideration should be given to the possibility of enhancing the resilience of parents, arguably even prior to their child’s death, such as by enhancing mindfulness, acceptance and psychological flexibility. Importantly, the Risk and Resilience Model of Parental Bereavement highlights the importance of considering both risk and resilience factors, and how these may, in combination, impact on bereavement outcomes.

A number of key issues warrant further research in order to better inform the development of evidence-based clinical interventions. If factors such as psychological flexibility and mindfulness are indeed associated with more favourable parental bereavement outcomes, how can these coping styles be taught? At what point should they be taught—before or after a child’s death? Would all parents facing bereavement benefit from these approaches, or is there a subset of parents who would receive most benefit? Notably, Bonanno [17] cautioned against assuming that there is a single resilience pathway. It may be that individuals with certain risk factors receive particular benefit from specific resilience factors that serve to compensate for the risk. More research is needed to address these questions.

## 11. Conclusions

The death of a child following a life-limiting illness is an incredibly stressful experience for parents, which may render these parents vulnerable to a range of adverse bereavement outcomes. The current paper sought to enhance understanding of a wide range of factors potentially impacting on parental bereavement outcomes following the death of a child due to a life-limiting condition. Studies have commonly focused on single risk factors and/or have been compromised by recruitment limitations and very small sample sizes, consequently resulting in some conflicting findings. These limitations are further heightened if the research lacks a theoretical framework. The Risk and Resilience Model of Parental Bereavement was proposed to enable consideration of a range of factors from within a broader theoretical framework. A better understanding of the complex interplay between various risk and resilience factors may allow health professionals to carry out comprehensive, holistic assessments prior to a child’s death, enabling them to identify parents who may be vulnerable to poorer bereavement outcomes. Further research is needed into interventions that heighten or promote resilience, particularly amongst parents identified as being at high risk of poor bereavement outcomes.
